# Supplementary material for: Translating genomic tools to Raman spectroscopy analysis enables high-dimensional tissue characterization on molecular resolution
Source: Nat Commun. 2023 Sep 19;14:5799. doi: 10.1038/s41467-023-41417-0 (PMC10509269; doi:10.1038/s41467-023-41417-0)
Supplement: Supplementary file 1 — Supplementary Information [file 41467_2023_41417_MOESM1_ESM.pdf]

Supplementary information to

Translating genomic tools to Raman spectroscopy analysis enables high-dimensional tissue characterization on molecular resolution

Manuel Sigle[1], Anne-Katrin Rohlfing[1], Martin Kenny[2], Sophia Scheuermann[3,4], Na Sun[5], Ulla Graeßner[3], Verena Haug[1], Jessica Sudmann[1], Christian Seitz[3,4], David Heinzmann[1], Katja Schenke-Layland[4,6,7], Patricia B Maguire[8], Axel Walch[5], Julia Marzi[4,6,7,#], Meinrad Paul Gawaz[1,#,*]

[1]Department of Cardiology and Angiology, University Hospital Tuebingen, Eberhard Karls University Tuebingen, 72076 Tuebingen, Germany
[2]School of Pharmacy and Biomolecular Sciences, Irish Centre for Vascular Biology, Royal College of Surgeons in Ireland, Dublin, Ireland.
[3]Department of Pediatric Hematology and Oncology, University Children's Hospital Tuebingen, 72076 Tuebingen, Germany
[4]Cluster of Excellence iFIT (EXC 2180) "Image-Guided and Functionally Instructed Tumor Therapies", University of Tuebingen, 72076 Tuebingen, Germany
[5]Research Unit Analytical Pathology, Helmholtz Zentrum Muenchen, German Research Center for Environmental Health (GmbH), Neuherberg, Germany
[6]Institute of Biomedical Engineering, Department for Medical Technologies and Regenerative Medicine, Eberhard Karls University Tuebingen, 72076 Tuebingen, Germany
[7]NMI Natural and Medical Sciences Institute at the University of Tuebingen, 72770 Reutlingen, Germany
[8]UCD Conway SPHERE Research Group, Conway Institute, University College Dublin, Dublin, Ireland; School of Biomolecular and Biomedical Science, University College Dublin, Dublin, Ireland; Institute for Discovery, O'Brien Centre for Science, University College Dublin, Dublin, Ireland.
[#]contributed equally

*corresponding author: Meinrad.Gawaz@med.uni-tuebingen.de

Table of content

Content	Description	Page
Supplementary Figure **S1**	Overview over technical and programmatic workflow.	3
Supplementary Figure **S2**	Impact of scaling, averaging and outlier removal on clustering results.	5
Supplementary Figure **S3**	Election of numbers of Principal Components and cluster resolution.	6
Supplementary Figure **S4**	Evaluation of intra-sample reproducibility.	7
Supplementary Figure **S5**	Spatially-aware clustering of whole spectrum and fingerprint spectrum data in comparison.	8
Supplementary Figure **S6**	Accuracy of fibrosis predictions using unsupervised clustering algorithms.	9
Supplementary Figure **S7**	Clustering results using different resolutions, dimensions, and methods.	10
Supplementary Figure **S8**	Molecular differences between myocardial subclusters identified by spatially-aware und spatially-unaware clustering.	11
Supplementary Figure **S9**	Inter-sample reproducibility and statistical analysis of remodeling and healthy myocardial subclusters.	13
Supplementary Figure **S10**	Inter-sample reproducibility and statistical comparison of myocardial infarction sections.	14
Supplementary Figure **S11**	Molecular dynamics along pseudotime trajectory to visualize transition from physiology to pathology.	15
Supplementary Figure **S12**	Spatial trajectories with molecular dynamics towards pathology.	16
Supplementary Figure **S13**	Raman-MALDI multi-omics.	17
Supplementary Figure **S14**	Alignment of Raman Brightfield Image and MACSima™ Autofluorescence Image	18
Supplementary Figure **S15**	Raman-MACSima™ Multi-Omics approach with paraffin sections.	19
Supplementary Figure **S16**	Assessment on accuracy of delineation of different cell types by Raman spectroscopy on tissue sections.	20
Supplementary Figure **S17**	Comparison of Raman spectromics against classical k-means clustering and deep-learning models	21
Supplementary Table **S1**	Characteristic Raman peaks and their assignment.	25
Supplementary Table **S2**	List of used antibodies for MACSima™ multicolor immuno-fluorescence staining.	26
References	References for Supplementary Information	27

Supplementary Figure S1

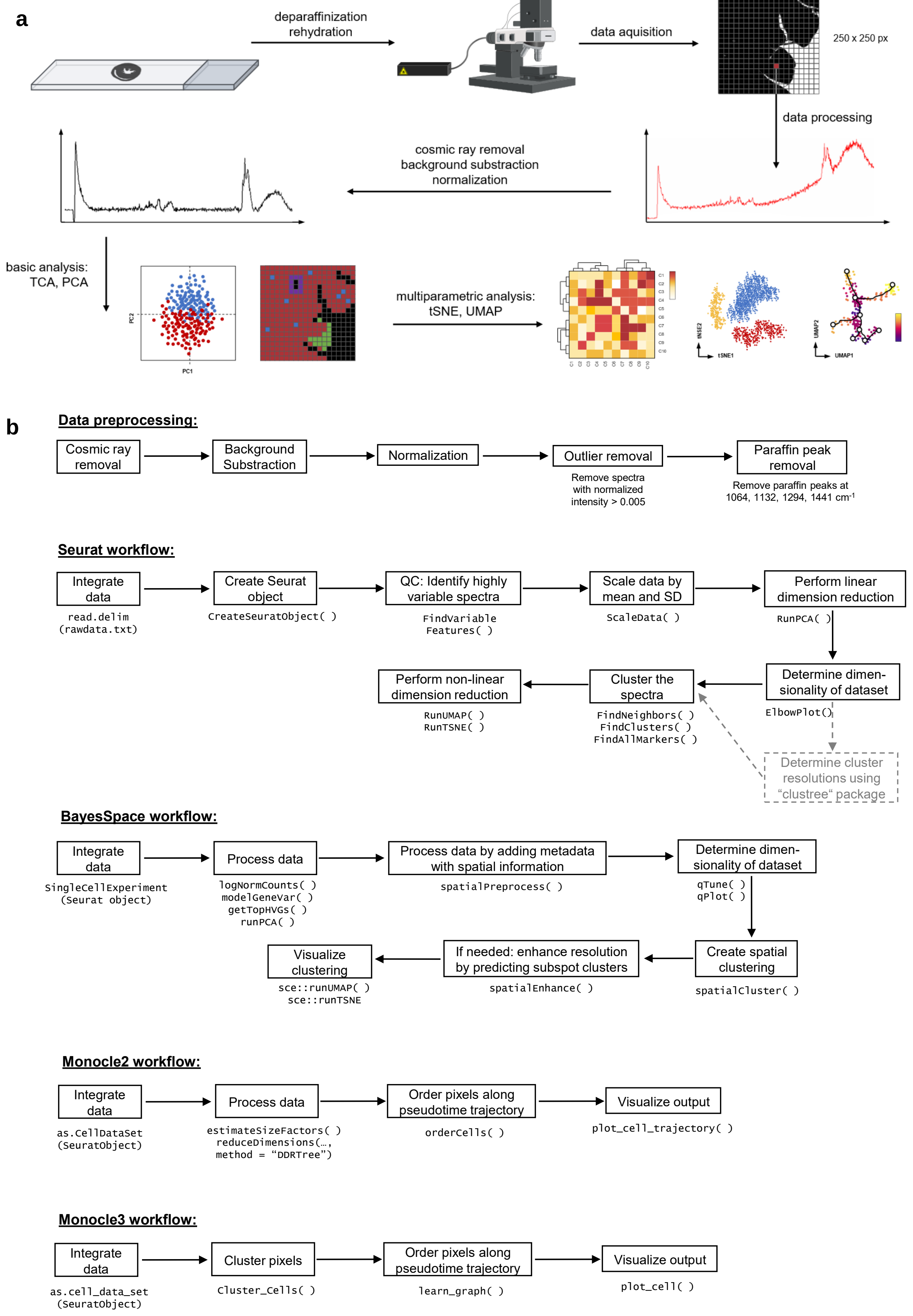

Supplementary Figure S1: Overview over technical and programmatic workflow.

a Technical workflow, starting with deparaffinization and rehydration of a FFPE tissue section, image aquisition by Raman microscope leading to a data matrix of 250x250 pixels; each pixel contains raw Raman wavenumbers and corresponding intensities; consecutive cosmic ray remodel, background correction and normalization of Raman data; subsequently basis analysis and following multiparametric analysis. **b** Programmatic workflow using mentioned R packages.

Supplementary Figure S2

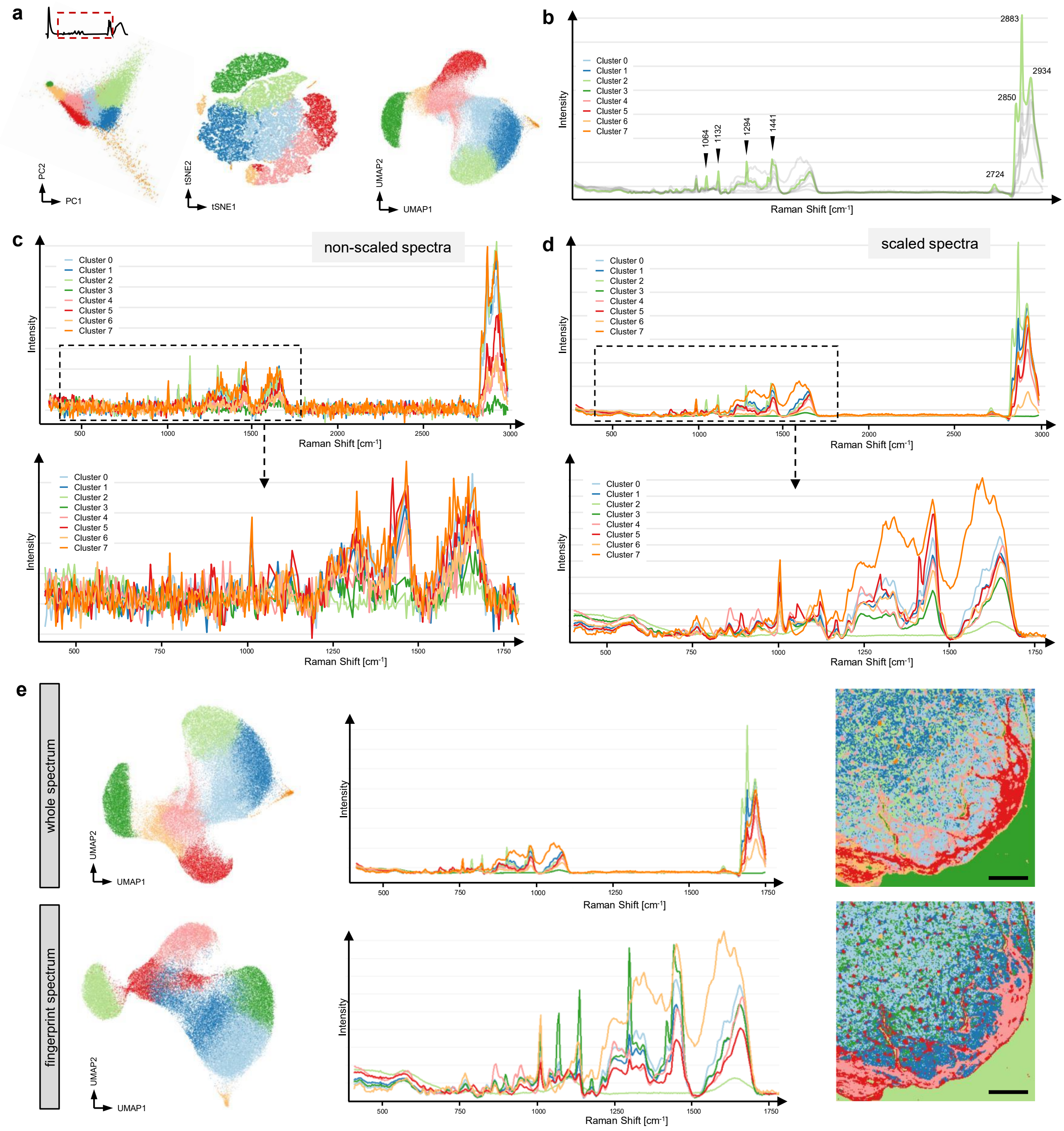

Supplementary Figure S2: Impact of scaling, averaging and outlier removal on clustering results. **a** Different projections of whole Raman spectrum acquired from the section of subendocardial fibrosis (Fig. 1d/e). **b** Cluster average spectrum with dedicated cluster of paraffin-rich pixels (green). Characteristic peaks are marked with black arrows. **c,d** Differences between manual averaging of raw data per cluster for whole and fingerprint spectrum (**c** top and bottom) versus Seurat-scaled and averaged cluster means (**d** top and bottom). **e** UAMP plot, average spectrum and cluster image of spatially-unaware Raman data without outlier removal and paraffin peak removal for whole spectrum (top) and fingerprint spectrum (bottom).

Supplementary Figure S3

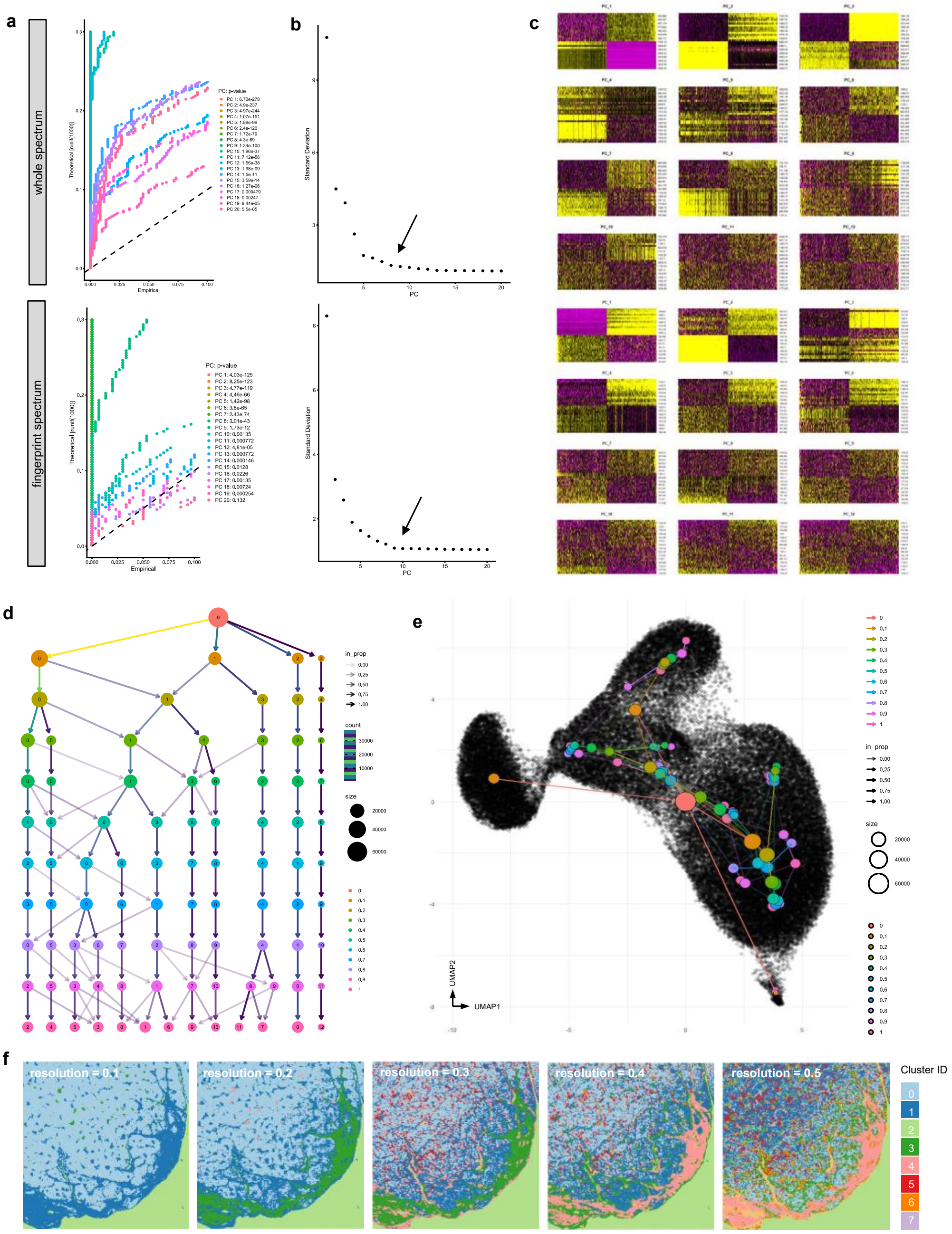

Supplementary Figure S3: Election of numbers of Principal Components and cluster resolution. **a** JackStraw analysis for PCA significance using whole spectrum (top) and fingerprint spectrum (bottom) data. **b** Elbow Plot with ranking of PCs based on the percentage of variance explained for whole (top) and fingerprint (bottom) spectrum. Number of PCs was selected at the 'elbow" of the plot. **c** Visualization of the top 12 PCs for whole (top) and fingerprint (bottom) spectrum. **d** Influence of different cluster resolutions on clustering results, analyzed using the "clustree" R package. As an example, selecting a resolution of 0.1 results in 4 clusters (second row, dark yellow). Increasing the resolution to 0.2, results in a split of cluster 1 into 2 subclusters. The 2 clusters to the left of the tree are that distinct, that changes in cluster resolution do not influence the value of k clusters. **e** Overlayed clustree analysis with spectra in UMAP projection, demonstrating the split of clusters as visualized by the tree, but here in UMAP projection. **f** Cluster images when using different resolutions.

Supplementary Figure S4

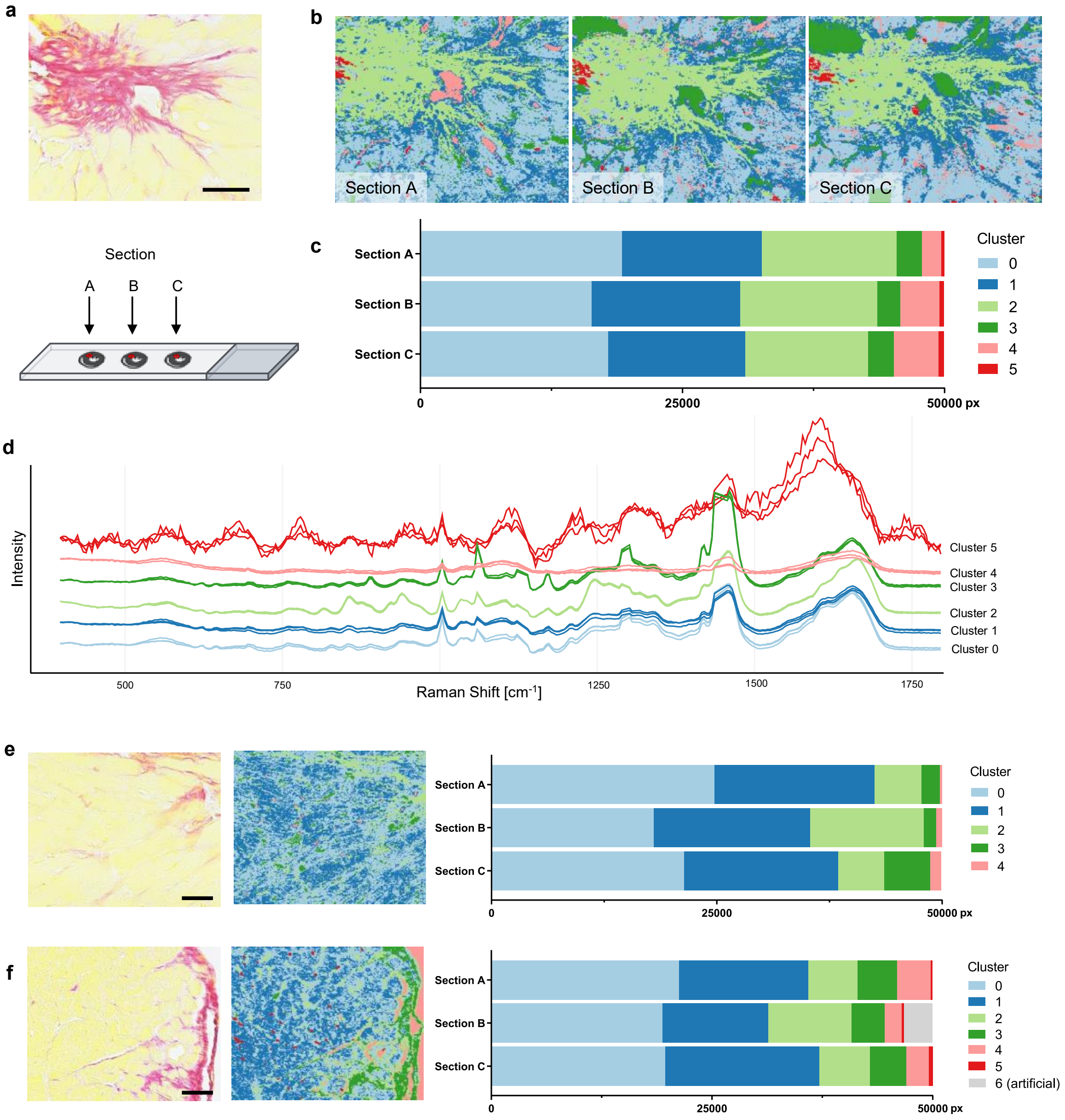

Supplementary Figure S4: Evaluation of intra-sample reproducibility.

a To evaluate the robustness of Raman Spectromics analyses, we performed sequential scans of n = 3 adjacent sections at the same spatial location. **b** Seurat-derived cluster analysis displays a visually comparable result. The pink cluster from Section A and the dark green cluster from B and C derive from paraffin residues on the slides. **c** Quantitative analysis of cluster sizes by direct comparison of the cluster proportions, underlining reproducibility in different sections. **d** Cluster average spectra found in all three samples. While Cluster 0 to 4 display a high fit, cluster 5 appears more heterogenous. Looking at the spatial distribution of the red cluster, one can consider it as artifact cluster. **e/f** Further sections of the control group (**e**) and another area of subendocardial fibrosis in the Ang II treatment group (**f**). Cluster proportions show comparable results. In section B from **Fig. 2f** an artificial cluster occurred from a tissue fold in the scan area.

Supplementary Figure S5

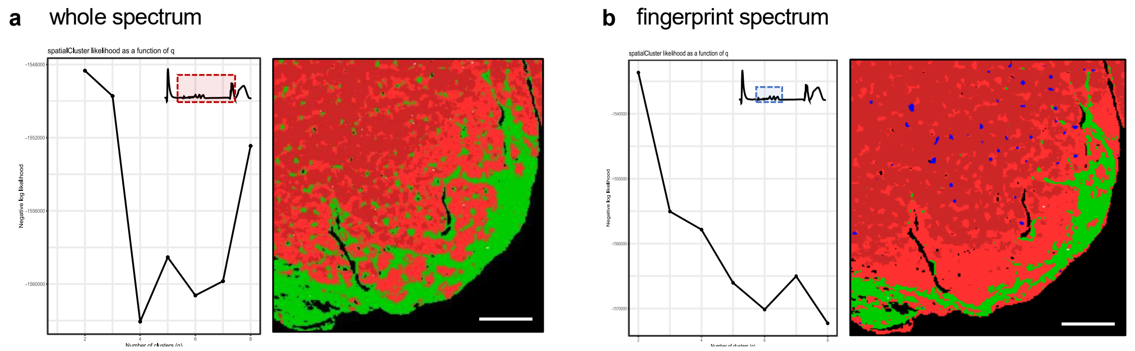

Supplementary Figure S5: Spatially-aware clustering of whole spectrum and fingerprint spectrum data in comparison.

a/b, left The number of clusters q was determined by the elbow of the pseudo-log-likelihood plot for whole (**a**) and fingerprint (**b**) spectrum. **a,b, right** Manually colored clusters found by unsupervised spatially-aware cluster analysis. Green denotes fibrosis cluster, red myocardium. Notably, restriction of the Raman spectrum to fingerprint spectrum (**b**) substantially improved cluster specificity for fibrosis.

Supplementary Figure S6

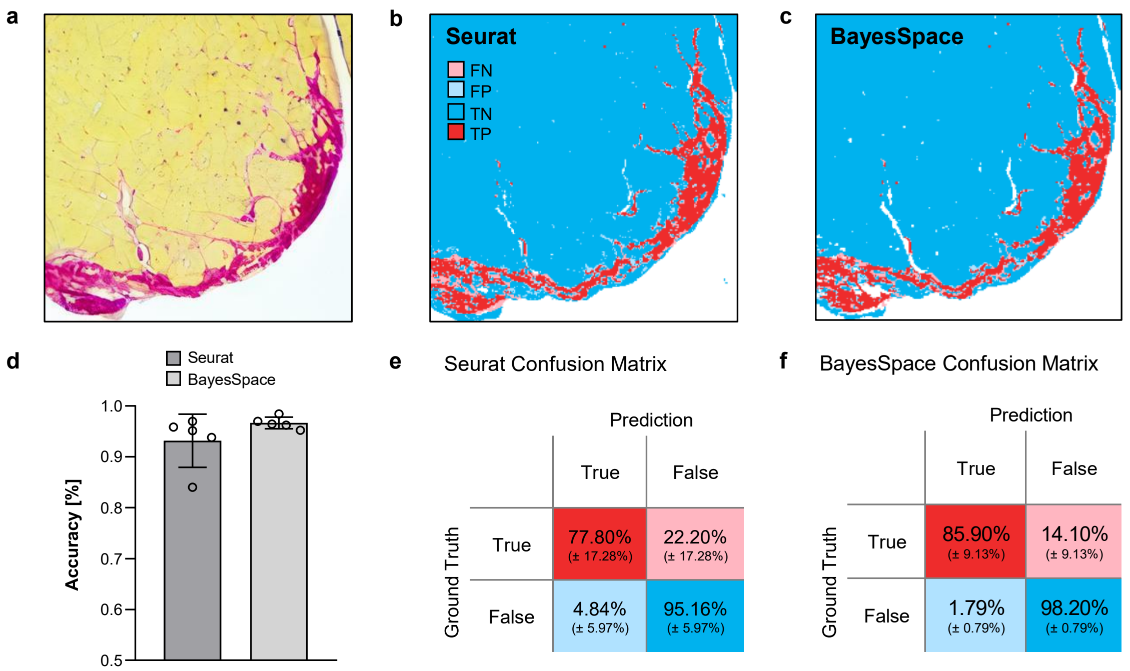

Supplementary Figure S6: Accuracy of fibrosis predictions using unsupervised clustering algorithms.

a Picrosirius red staining used as ground truth for fibrotic areas. **b** Prediction of fibrotic areas by Seurat. Dark colors represent true positive (TN, dark red) or true negative (TN, dark blue) predictions, while light colors reflect false negative (FN, light red) or false positive (FP, light blue) assignments. **c** Prediction of fibrotic areas by BayesSpace. Overall, both classifications look quite similar and accurate. **d** Quantification by calculation of the accuracy of fibrosis prediction. BayesSpace outperforms Seurat and is more robust than Seurat's classification. **e/f** Confusion matrix of Seurat's (left) and BayesSpace's (right) predictions, by calculating the mean rates (± standard deviation) of prediction. The true positive rate (dark red) is also known as sensitivity, and the true false rate (dark blue) is the specificity. BayesSpace provides higher sensitivity and specificity in prediction of fibrotic clusters, or overall cluster assignments.

Supplementary Figures S7

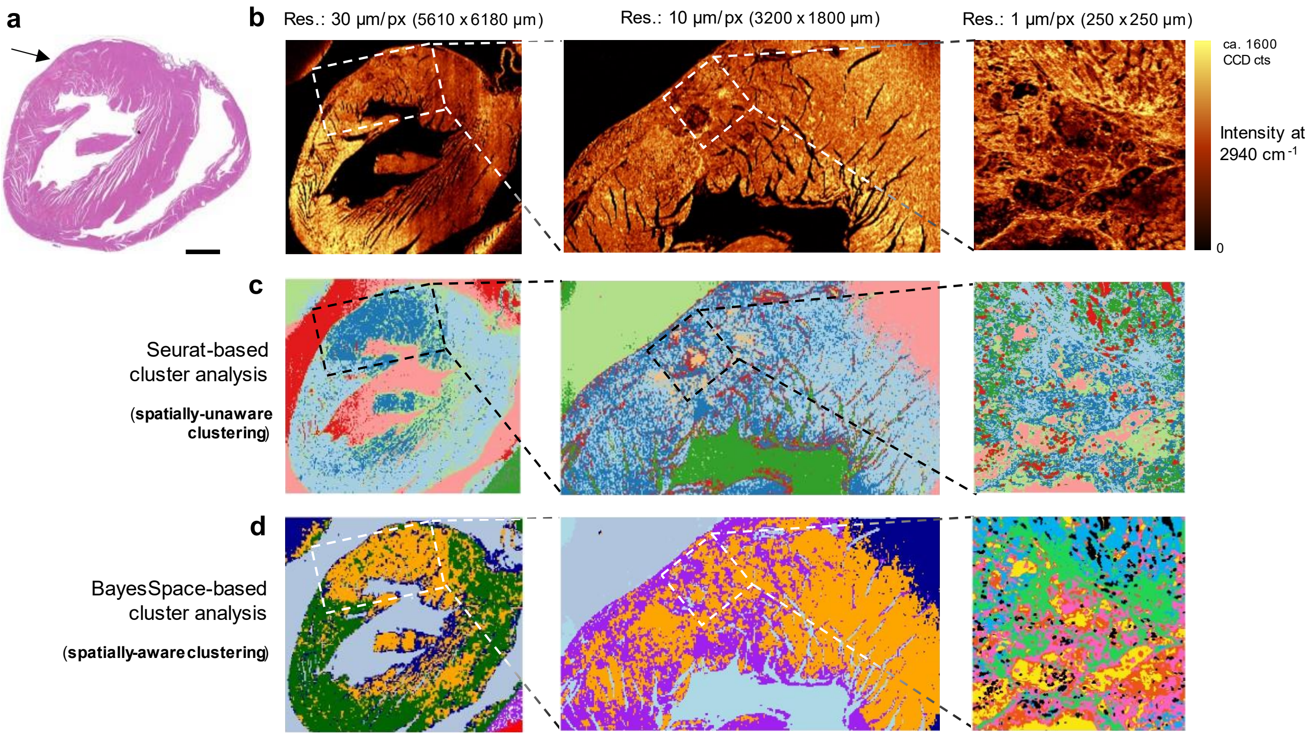

Supplementary Figure S7: Clustering results using different resolutions, dimensions, and methods.

a H&E staining of a section of murine acute myocardial infarction. Arrow points to ligation site. **b** Low resolution/large area scan of the whole heart, medium resolution/medium area scan of the infarct area and high resolution/small area scan of the infarct border. Intensity-based image at 2940 cm^{-1}. **c** Seurat-based cluster analysis (spatially-unaware clustering) of the three scans. **d** BayesSpace based cluster analysis (spatially-aware clustering).

Supplementary Figures S8

a

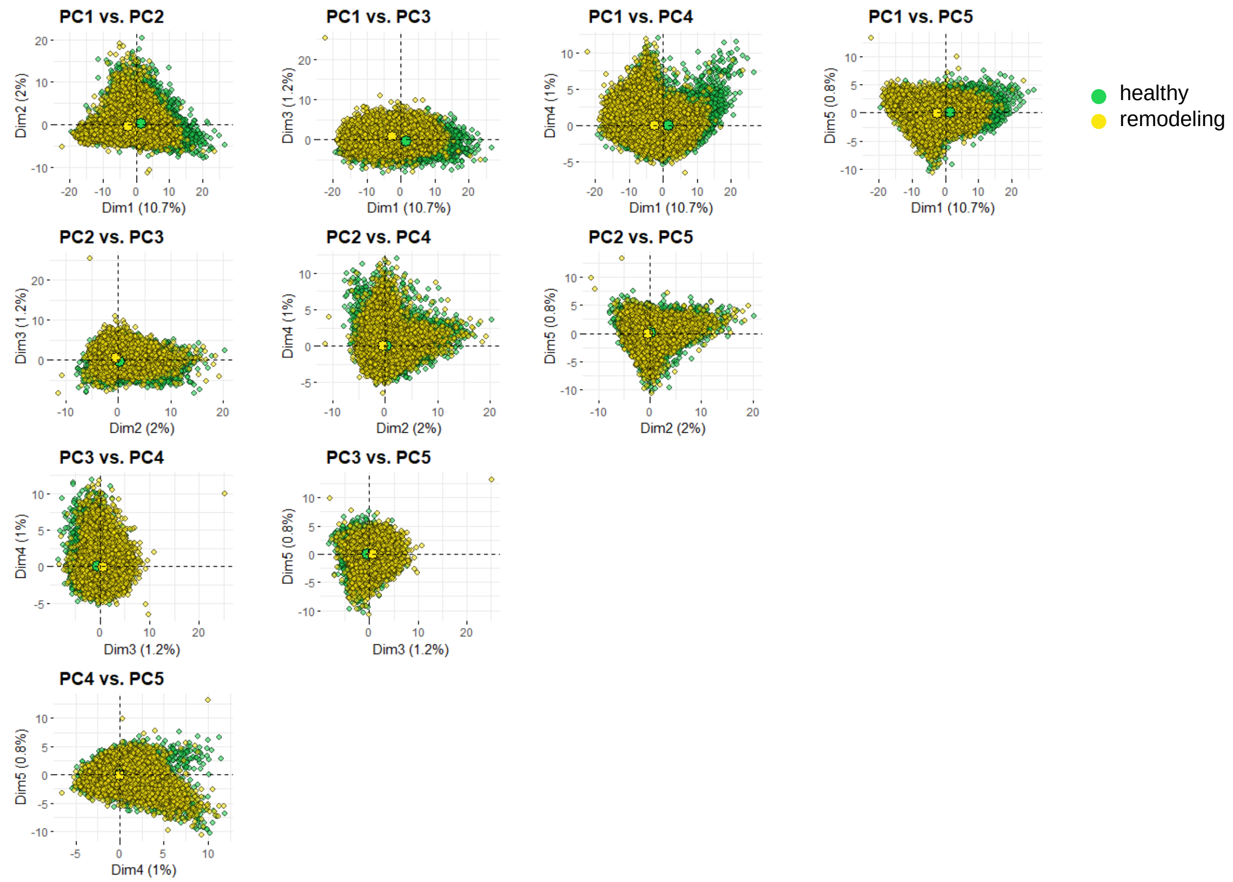

b

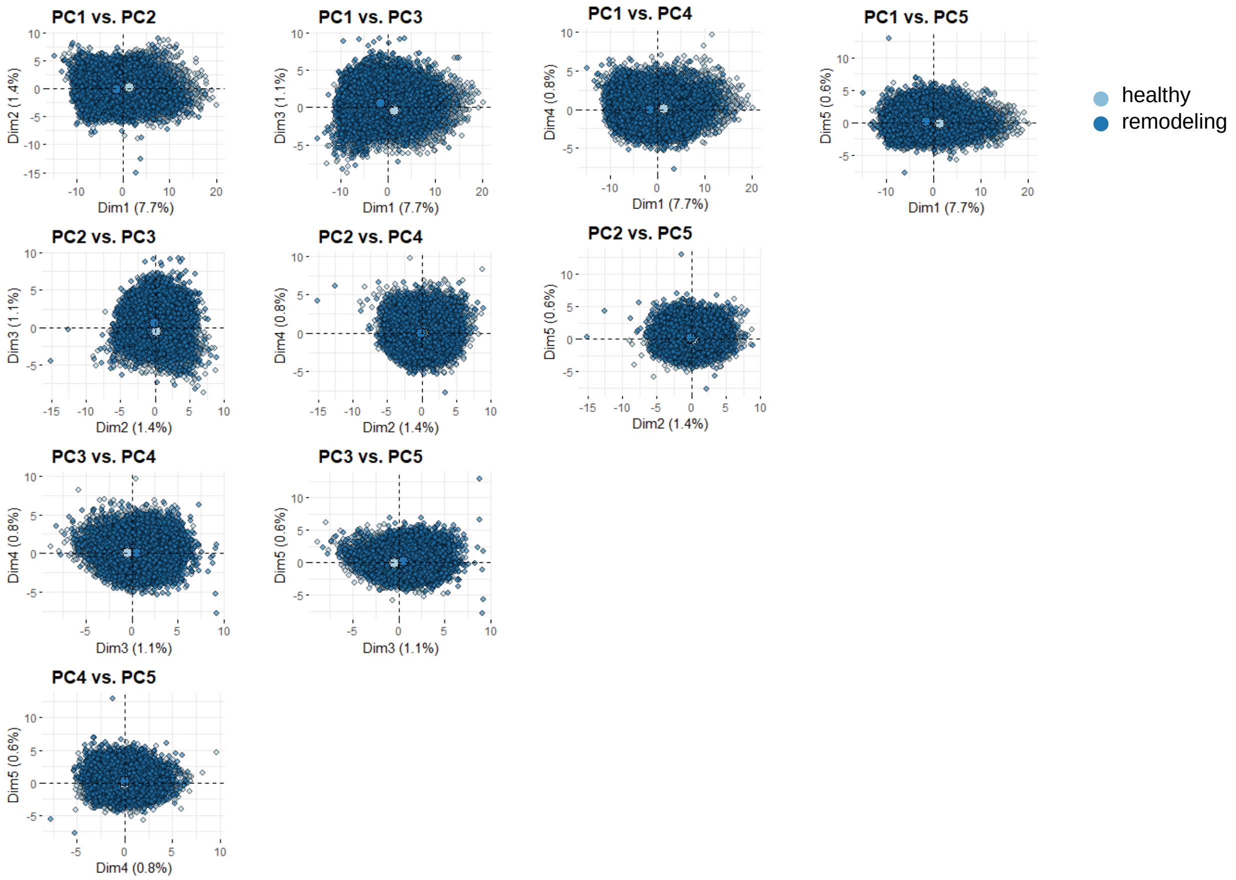

c

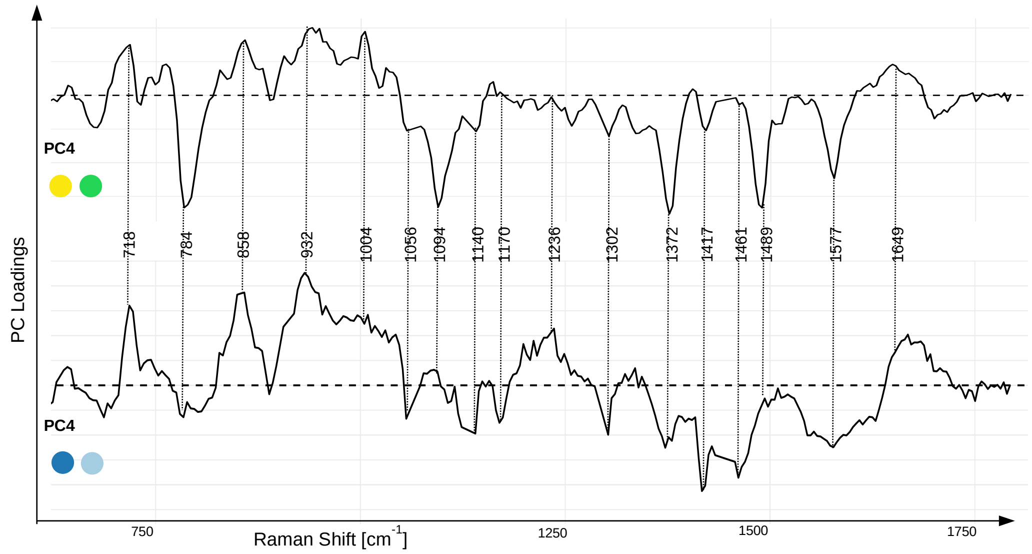

Supplementary Figure S8: **Molecular differences between myocardial subclusters identified by spatially-aware und spatially-unaware clustering.**

a Overview over the first 5 Principal Components when comparing myocardial subclusters identified by spatially-aware clustering (BayesSpace). **b** Overview over the first 5 Principal Components when comparing myocardial subclusters identified by spatially-unaware clustering (Seurat). **c** PC Loadings for PC4.

Supplementary Figures S9

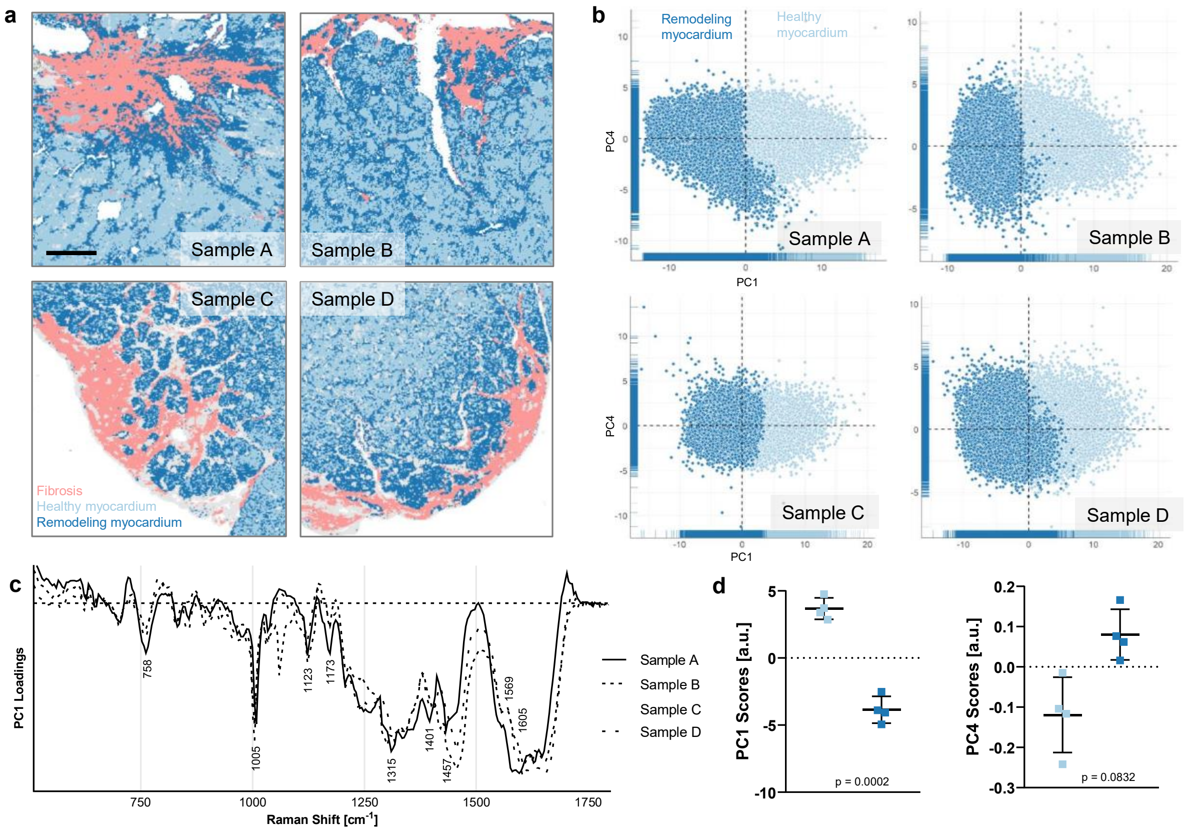

Supplementary Figure S9: **Inter-sample reproducibility and statistical analysis of remodeling and healthy myocardial subclusters.**

a Cluster images of 4 individual hearts with areas of fibrosis and surround area of remodeling myocardium. Pink denotes the fibrotic cluster; light blue is interpreted as healthy and dark blue remodeling myocardium. **b** Principal Component Analysis (PCA) of the clusters found by unsupervised cluster analysis in a. Spectra for healthy and remodeling myocardium show a strong separation into 2 clusters. **c** Loadings plot for Principal Component 1 (PC1). All 4 samples show comparable spectral and hence molecular differences leading to two distrinct myocardial subclusters. **d** PC Scores plot for PC1 and PC. PC1 displays highly significant differences between healthy and remodeling myocardium (p = 0.0002), PC4 is nearly significant (p = 0.0832). n = 4 from 4 individual mice. Student's two-sided paired t-test. Plotted is mean ± SEM.

Supplementary Figures S10

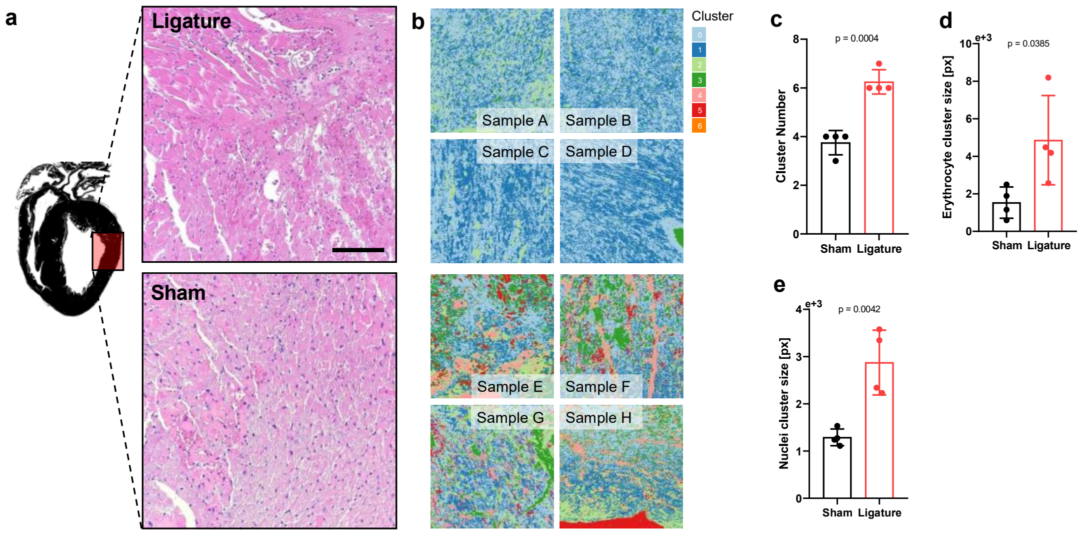

Supplementary Figure S10: **Inter-sample reproducibility and statistical comparison of myocardial infarction sections.**

a Representative H&E Stainings of heart regions in close neighborhood of the ligature (top) or sham-operated region. **b** Cluster images determined by unsupervised cluster analysis based on molecular composition of the sample. **c** Quantification of the cluster number found by Seurat's clustering algorithm. **d** Quantification of the cluster size of clusters resulting from erythrocytes and their characteristic hemine peaks. **e** Quantification of the cluster size of clusters resulting from characteristic peaks for RNA/DNA. Student's unpaired t-test. Bar plots are mean ± SEM.

Supplementary Figure S11

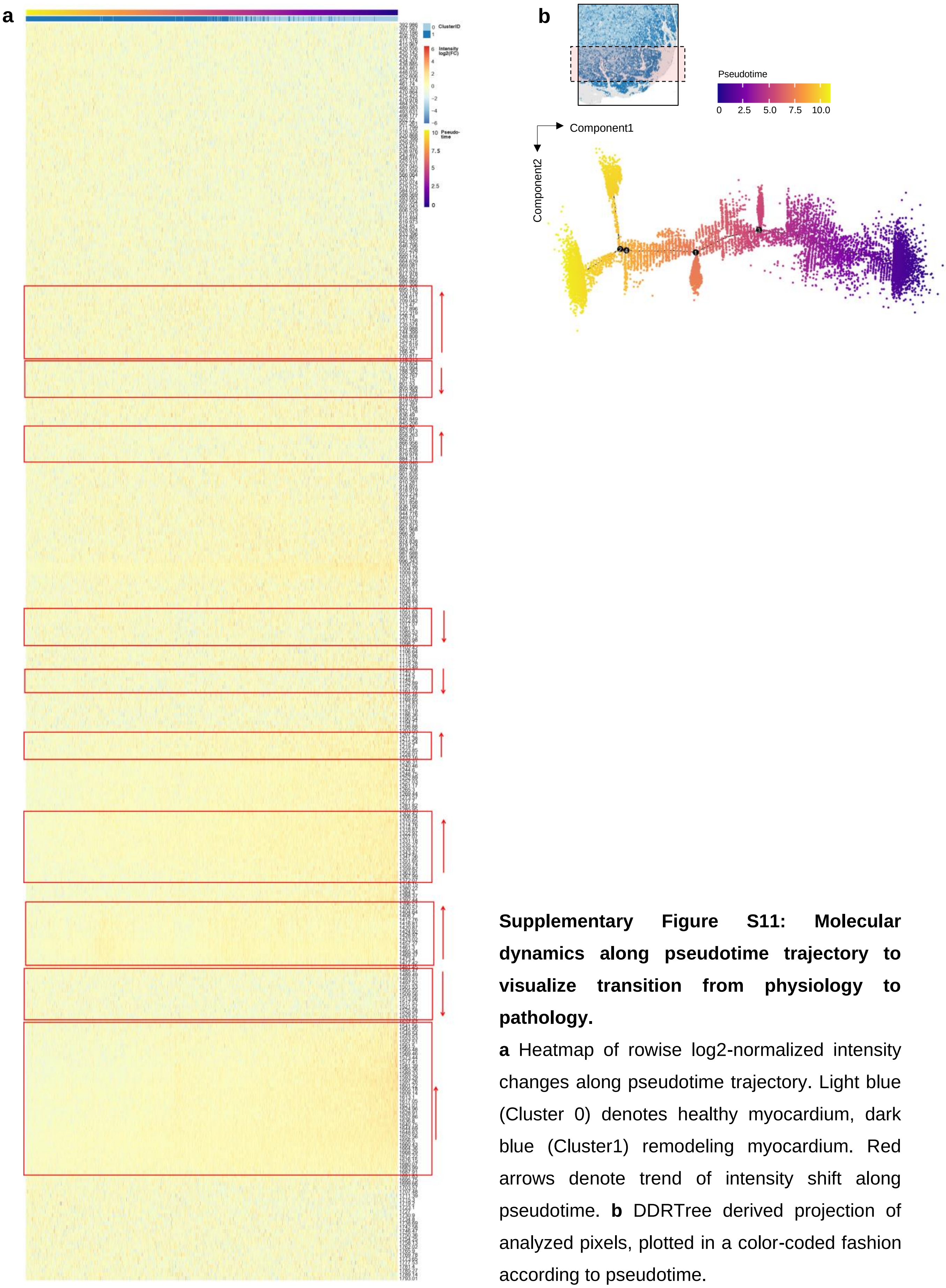

Supplementary Figure S11: Molecular dynamics along pseudotime trajectory to visualize transition from physiology to pathology.

a Heatmap of rowise log2-normalized intensity changes along pseudotime trajectory. Light blue (Cluster 0) denotes healthy myocardium, dark blue (Cluster1) remodeling myocardium. Red arrows denote trend of intensity shift along pseudotime. **b** DDRTree derived projection of analyzed pixels, plotted in a color-coded fashion according to pseudotime.

Supplementary Figure S12

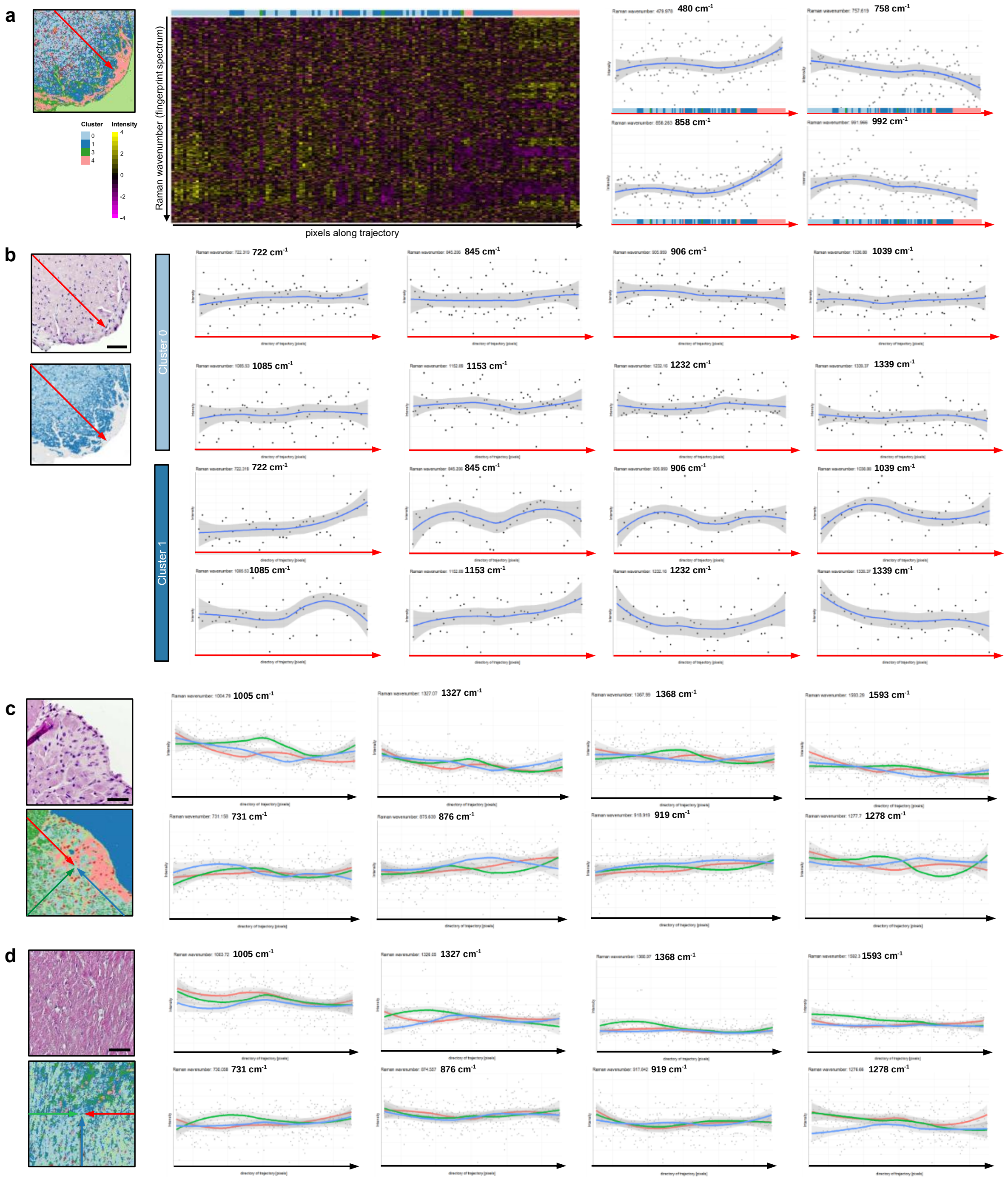

Supplementary Figure S12: Spatial trajectories with molecular dynamics towards pathology. **a** Heatmap of log2-normalized intensity changes in Raman fingerprint spectrum along red trajectory and selected wavenumbers and their dynamics when spatially approaching subpapillary fibrosis. **b** Intra-cluster heterogeneity and dynamics when comparing same Raman wavenumbers of pixels assigned to cluster 0 (top) to cluster 1 (bottom). Notably, cluster 0 (healthy myocardium) shows remarkably less dynamics in comparison to cluster 1 (remodeling myocard). **c** Validation of spatial trajectories by spatial approach to pathology from three cardinal points. **d** No or less dynamics are observed when looking at a healthy section.

Supplementary Figure S13

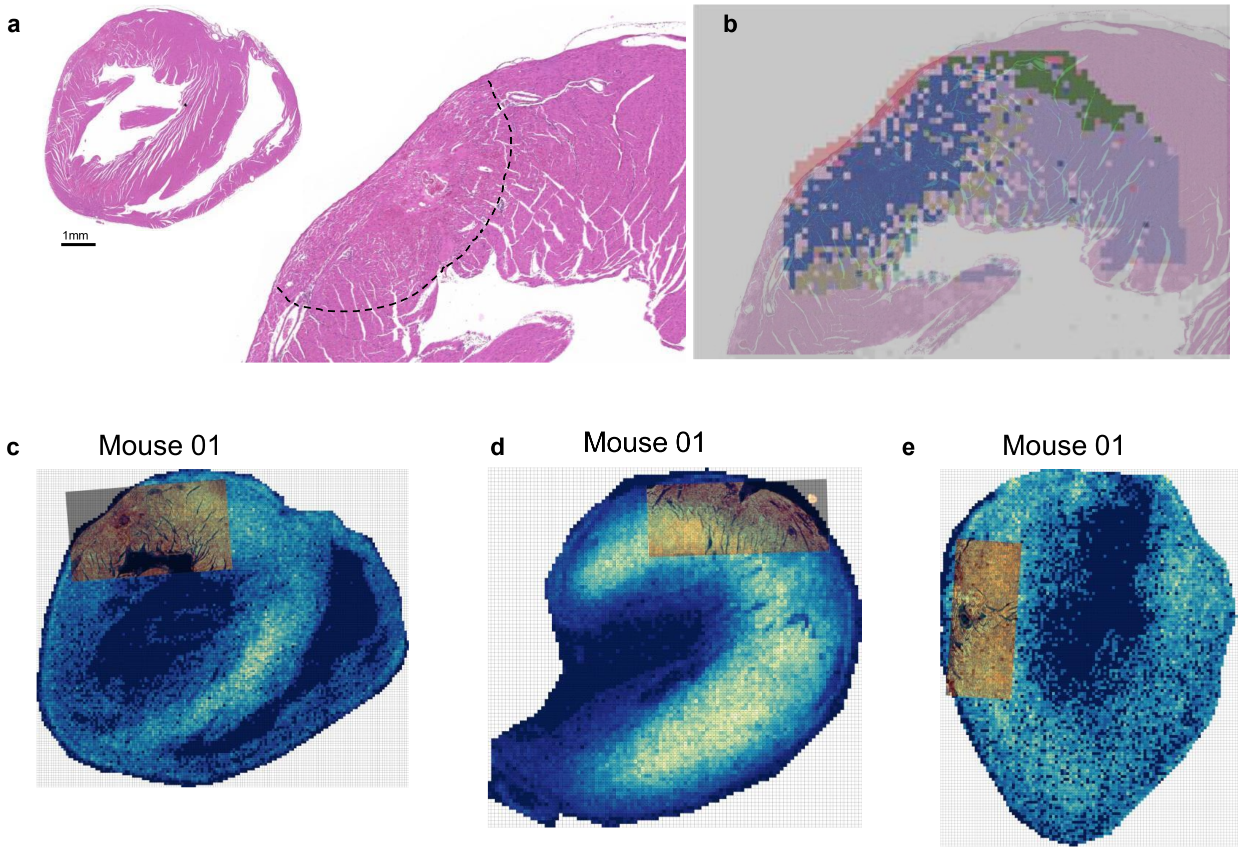

Supplementary Figure S13: Raman-MALDI multi-omics. a H&E staining of the section after MALDI analysis. The H&E stainings were used as ground truth imaged to verify location of remote and I/R border region clusters. **b** Clusters identified by Raman-MALDI multimodal cluster analysis. The dark blue cluster is clearly located inside the infarct region, while the light blue cluster corresponds the healthy myocardium. **c-e** Overlay images of rasterized MALDI images (m/z peak exemplary for Glucose-6-Phosphate) and Raman scans (Intensity exemplary at 2940 cm^{-1}).

Supplementary Figure S14

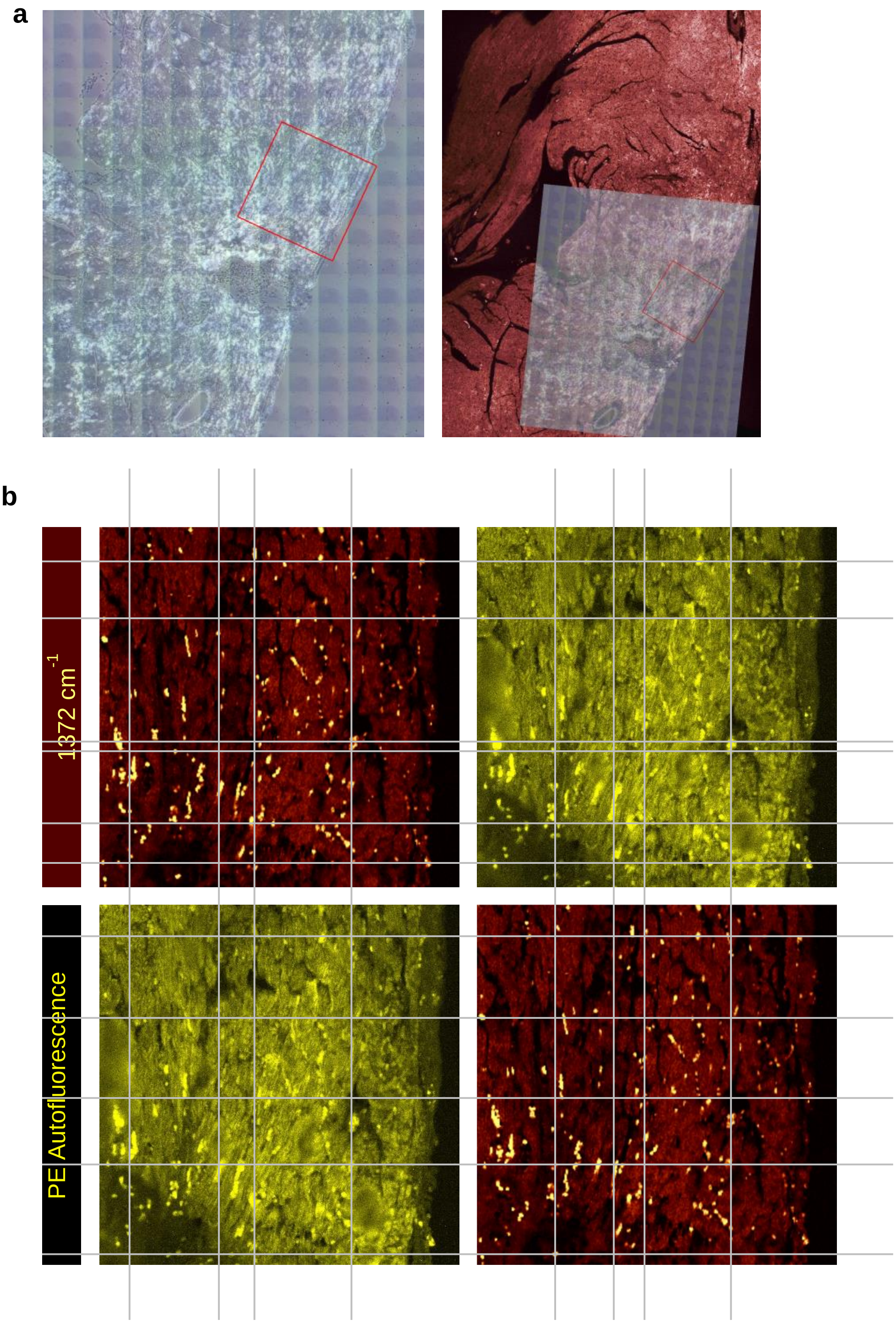

Supplementary Figure S14: Alignment of Raman Brightfield Image and MACSima^{TM} Autofluorescence Image

a Alignment of Raman Brightfield Image (left) and MACSima^{TM} Autofluorescence Image (right). The area with red borders marks the Raman scan area (300 x 300 pixels). **b** Erythrocytes produce strong autofluorescence in immunofluorescence images and Raman spectroscopy due to pyrrole ring vibrations of hemin. This fact was used to spatially align both images.

Supplementary Figure S15

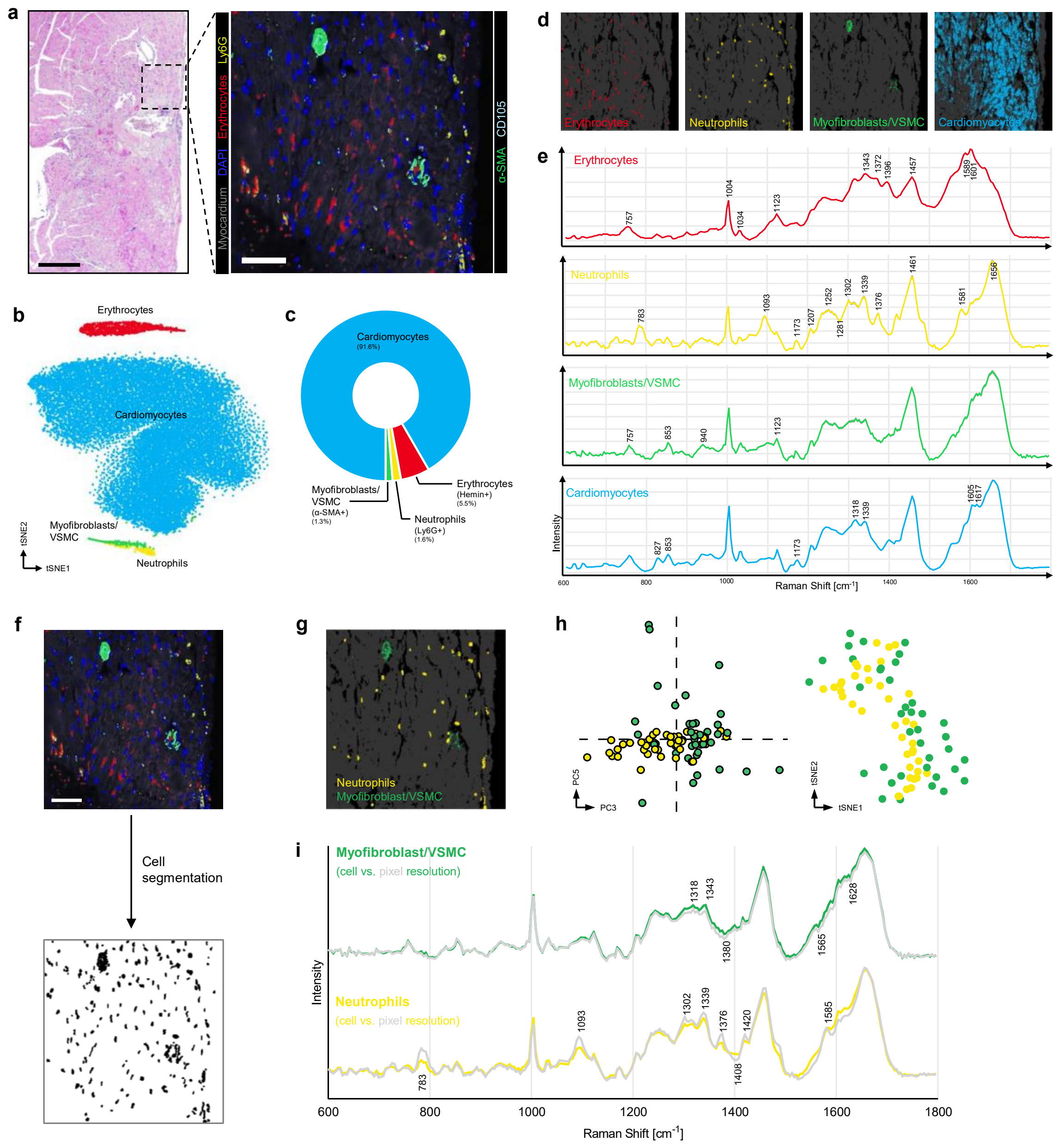

Supplementary Figure S15: Raman-MACSima™ Multi-Omics approach with paraffin sections.

a H&E and corresponding multiplexed immunofluorescence staining of murine myocardial infarction subjected to analysis. Scalebar 300 µm. **b** tSNE plot after dimensionality reduction of Raman spectra of identified cells. **c** Donut chart showing frequency distribution of identified cells (percent from absolute number of analyzed number of pixels). **e** Average spectra for erythrocytes (red), neutrophils (yellow), vascular cells (green) and cardiomyocytes (blue), together with their spatial representation. Scalebar 50 µm. VSMC: vascular smooth muscle cell. **f** Testing of another approach, where cells were segmented from the immunofluorescence image and at first spectra for each cell were averaged and then subjected to multidimensional analysis. **g** Neutrophils and vascular cells were chosen for this analysis. **h** Linear (PCA) and non-linear (tSNE) dimension reduction show a week separation into clusters for both cell types. **i** Average spectra from cell (colored) vs. pixel (in grey) resolution. Main differences between both spectra are labeled with the corresponding wavenumber. Differences are the result of outlier spectra which have been merged into actual correct spectra.

Supplementary Figure S16

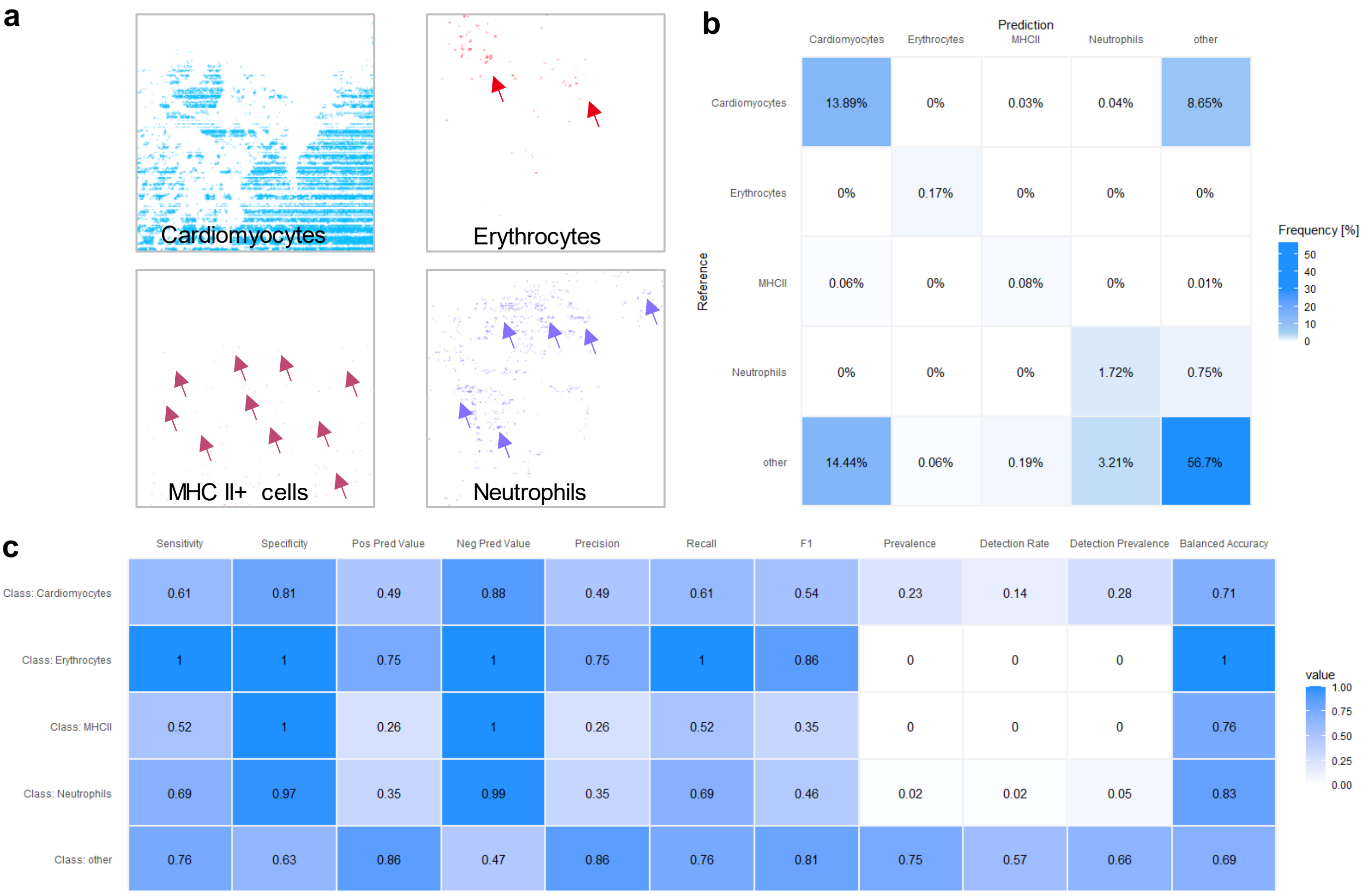

Supplementary Figure S16: Assessment on accuracy of delineation of different cell types by Raman spectroscopy on tissue sections.
a Cell-type specific average spectra were used as “reference spectra” to calculate the similarity to all spectra of a Raman scan. Spectra with a similarity > 0.99 were colored. **b** Overview over prediction and ground truth assignment of pixels to specific cell types. **c** Comprehensive overview of classification metrics.

Supplementary Figure S17

Supplementary Figure S17: Comparison of Raman spectromics against classical k-means clustering and deep-learning models.

a Quantitative comparison of the average intersection over union (IoU) between Raman spectromics derived clusters and those identified by k-means or deep-learning. **b** Cluster images from all three methods (Raman spectromics vs. k-means vs- deep learning (LSTM autoencoder, U-net with LSTM, U-net without LSTM).

a

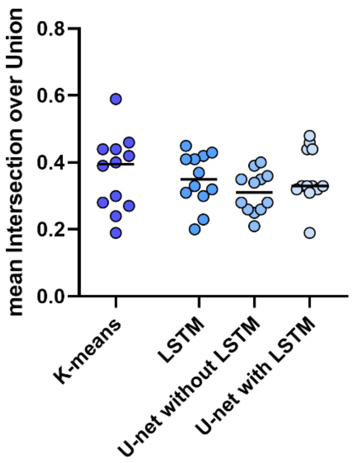

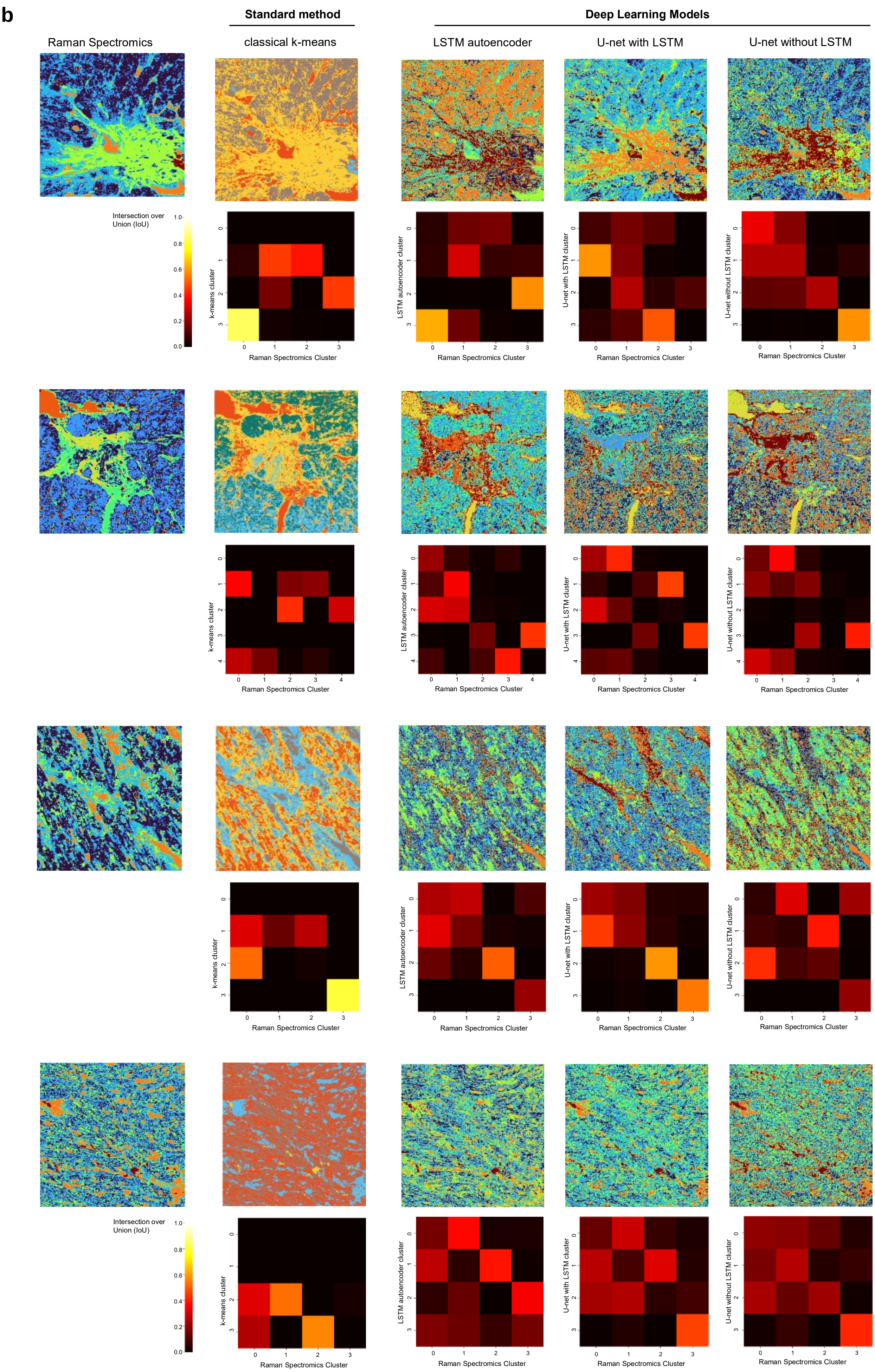
b
Standard method
Deep Learning Models
Raman Spectromics
classical k-means
LSTM autoencoder
U-net with LSTM
U-net without LSTM
Intersection over Union (IoU)
k-means cluster
LSTM autoencoder cluster
U-net with LSTM cluster
U-net without LSTM cluster
Raman Spectromics Cluster

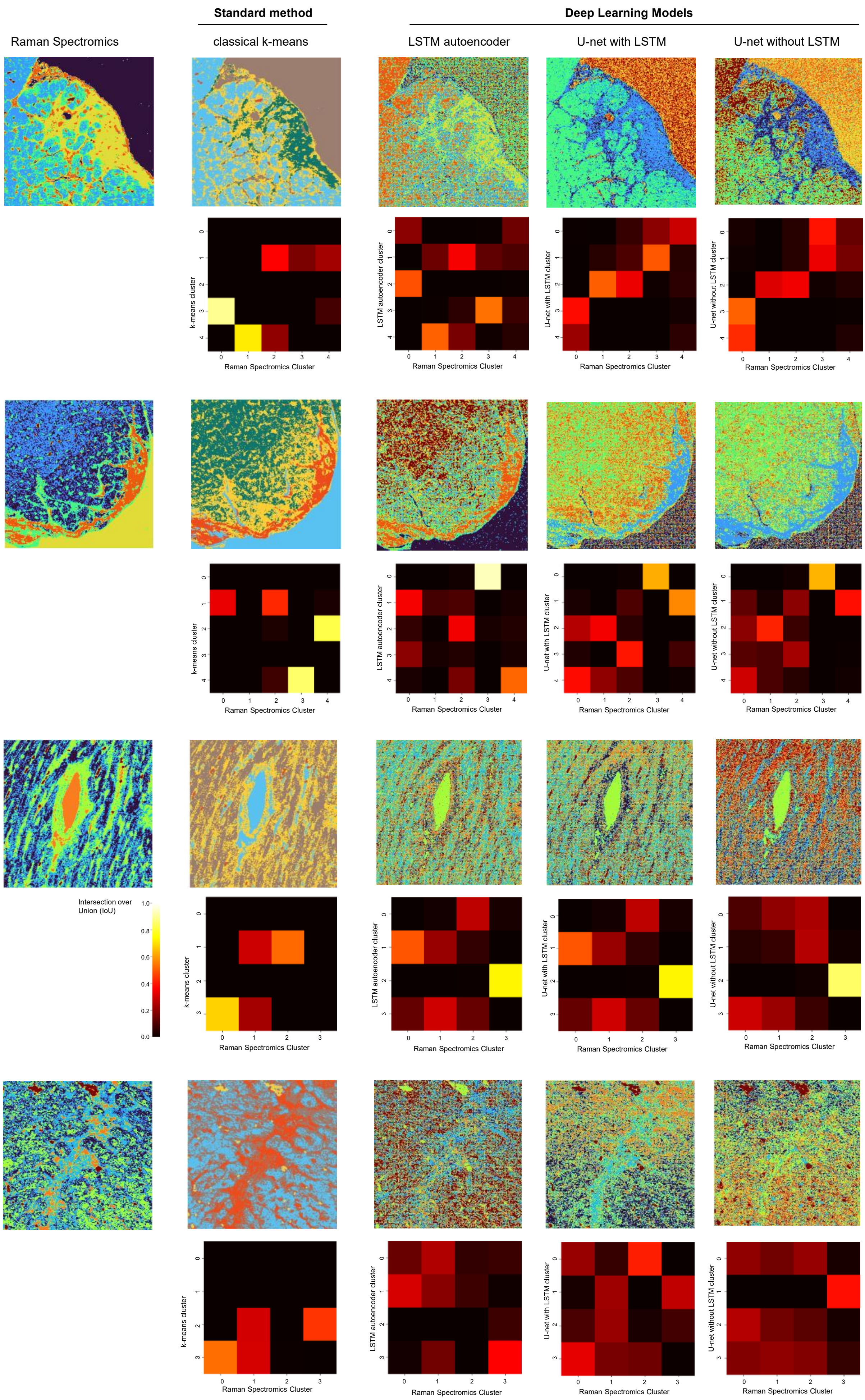
Standard method
Deep Learning Models
Raman Spectromics
classical k-means
LSTM autoencoder
U-net with LSTM
U-net without LSTM
k-means cluster
LSTM autoencoder cluster
U-net with LSTM cluster
U-net without LSTM cluster
Raman Spectromics Cluster
Intersection over Union (IoU)
1.0
0.8
0.6
0.4
0.2
0.0

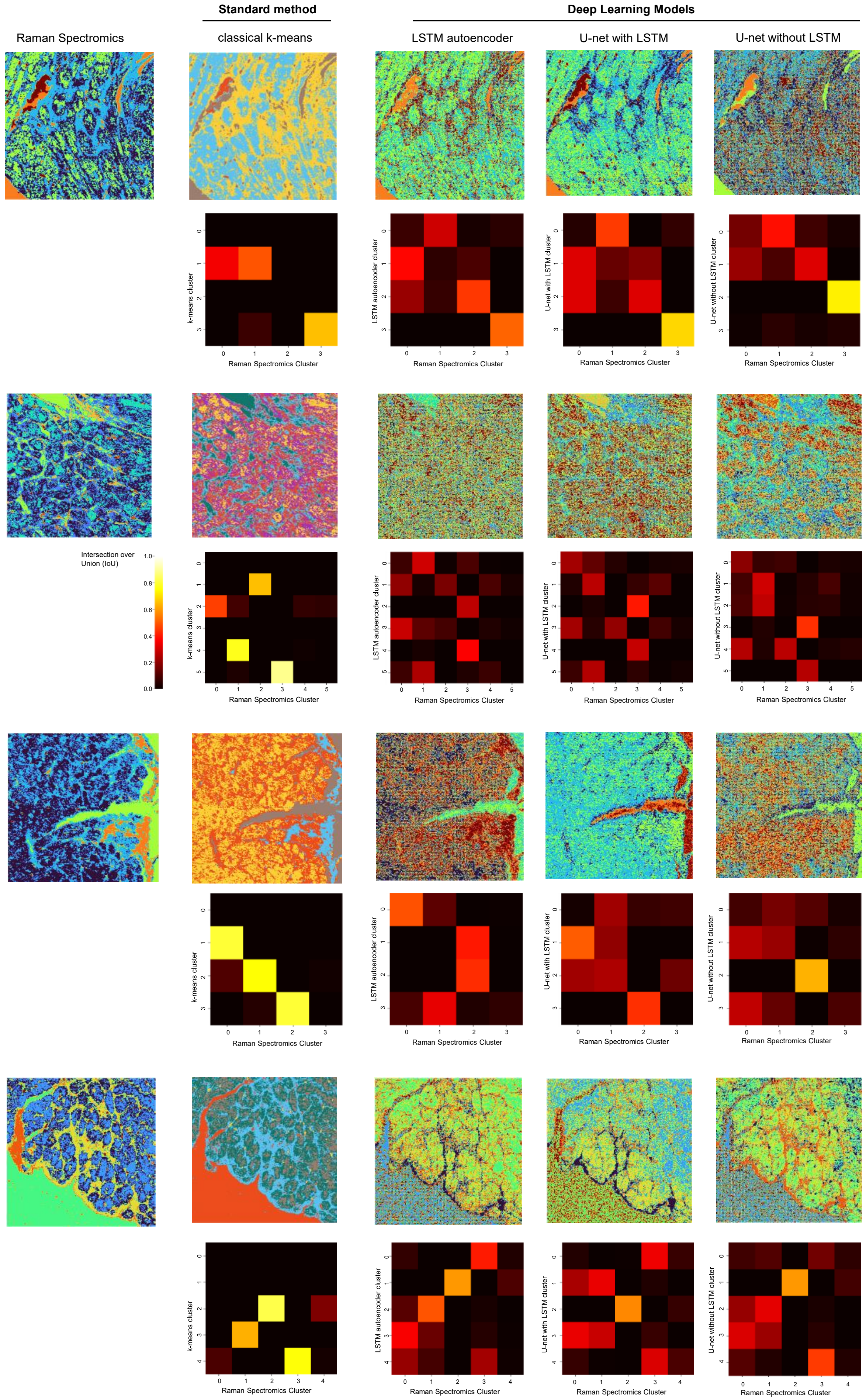
Standard method
Deep Learning Models
Raman Spectromics
classical k-means
LSTM autoencoder
U-net with LSTM
U-net without LSTM
k-means cluster
LSTM autoencoder cluster
U-net with LSTM cluster
U-net without LSTM cluster
Raman Spectromics Cluster
Intersection over Union (IoU)
1.0
0.8
0.6
0.4
0.2
0.0

Supplementary Table S1: Characteristic Raman peaks and their assignment

Raman Shift [cm^{-1}]	Molecular assignment	Structure
560	Si-O bending	Glass
722	O-C-N bending	Proteins[32]
757	Tryptophane or pyrrole ring breathing	Proteins[32], hemoglobin[2]
783-793	nucleic acids	DNA[24,25]
827	CH_3 stretch, tyrosine	Myosin[2]
853	C-C stretch	Myosin[2]
858	C-O vibrations, (Hydroxy-)Proline	Proteins, collagen[23]
889	CH_2 vibration	Paraffin[47]
940	skeletal vibration (C-C)	Proteins, collagen[23,37]
1000		Phenylalanine, NADH[35,36]
1005	ring breathing, Phenylalanine	Proteins[3]
1034	C-H in plane bending	Proteins[3]
1064	skeletal vibration (C-C)	Paraffin[47]
1070	C-C stretching	Carbohydrates, glucose[8,35]
1093-1096	Phosphodioxy-	DNA[24,4]
1100	Si-O-Si stretching	glass
1117	C-O stretching	Carbohydrates, glucose[37,35]
1123	C-O stretching	Carbohydrates, glucose[37,35]
1130	C-C stretch	Lipids, Paraffin[47]
1245-1252	Amide III	Proteins[32], collagen[23]
1301	CH_2 deformation	Paraffin[47]
1314-1319	Cytochrome c	Cytochrome c[12]
1339-1343	CH_2/CH_3 bending, C-C stretching tryptophane, pyrrole	Proteins[32], erythrocytes[2]
1372-1376	guanine	DNA[24,25]
1401	pyrrole	Erythrocytes[1]
1420	CH_3 deformation	Paraffin[47]
1444	CH_2/CH_3 deformation	Paraffin[47]
1457-1461	CH_2/CH_3 deformation	Proteins[32]
1569	CN & NH stretching	Proteins[32]
1580-1581	Cytosine, guanine	DNA[24,25]
1589	C=C stretching, porphyrin	Erythrocytes[1]
1605	C=C stretching	Phenylalanine[32], erythrocytes[1]
1618	v(C=C)	NADH[35,36]
1637	C-C asymmetric stretch	Erythrocytes[1]
1650-1680	Amide I	Proteins[32]
1678		NADH[35,36]

Supplementary Table S2: List of used antibodies for MACSima^{TM} multicolor immuno-fluorescence staining.

Target/Reagent	Clone	Fluorochrome	Dilution	Order No.	Supplier
Alpha-Smooth Muscle Actin	1A4	Alexa Fluor™ 488	1:50	53-9760-82	Thermo Fisher Scientific
Beta Actin	REAL1032	PE	1:50	130-127-405	Miltenyi Biotec
CD105	MJ7-18	FITC	1:50	130-102-915	Miltenyi Biotec
CD11b	M1-70-15-11-5	FITC	1:50	130-113-796	Miltenyi Biotec
CD11c	N418	FITC	1:50	130-122-939	Miltenyi Biotec
CD2	REA959	FITC	1:50	130-115-958	Miltenyi Biotec
CD3	REA641	FITC	1:50	130-119-798	Miltenyi Biotec
CD31	REAL260	PE	1:50	130-118-936	Miltenyi Biotec
CD4	REA604	PE	1:50	130-116-509	Miltenyi Biotec
CD41	REA1194	PE	1:50	130-122-760	Miltenyi Biotec
CD44	REA664	PE	1:50	130-118-694	Miltenyi Biotec
CD45	REA737	FITC	1:50	130-110-796	Miltenyi Biotec
CD61	REA1192	PE	1:50	130-122-148	Miltenyi Biotec
CD68	REA835	FITC	1:50	130-112-855	Miltenyi Biotec
CD80	REA983	FITC	1:50	130-116-459	Miltenyi Biotec
CD8b	REA793	FITC	1:50	130-111-710	Miltenyi Biotec
Cardiac Troponin T	REA400	PE	1:50	130-120-405	Miltenyi Biotec
Cytokeratin 19	A-3	PE	1:50	sc376126PE	Santa Cruz Biotechnology
DAPI Staining Solution	-	DAPI	1:100	130-111-570	Miltenyi Biotec
F4/80	REA126	FITC	1:50	130-117-509	Miltenyi Biotec
Ki67	REA183	FITC	1:50	130-117-691	Miltenyi Biotec
Ly-6G	REA526	FITC	1:50	130-120-820	Miltenyi Biotec
MHC Class II	REA813	FITC	1:50	130-112-386	Miltenyi Biotec
Vimentin	E-5	FITC	1:200	sc-373717FITC	Santa Cruz Biotechnology

References

1 Wood, B. R. & McNaughton, D. Raman excitation wavelength investigation of single red blood cells in vivo. *Journal of Raman Spectroscopy* **33**, 517-523 (2002).

2 Pascut, F. C. *et al.* Noninvasive detection and imaging of molecular markers in live cardiomyocytes derived from human embryonic stem cells. *Biophysical journal* **100**, 251-259 (2011).

3 Puppels, G. J., Garritsen, H. S., Segers-Nolten, G. M., de Mul, F. F. & Greve, J. Raman microspectroscopic approach to the study of human granulocytes. *Biophysical Journal* **60**, 1046-1056, doi:https://doi.org/10.1016/S0006-3495(91)82142-7 (1991).
