# Supplementary material for: Translating genomic tools to Raman spectroscopy analysis enables high-dimensional tissue characterization on molecular resolution
Source: Nat Commun. 2023 Sep 19;14:5799. doi: 10.1038/s41467-023-41417-0 (PMC10509269; doi:10.1038/s41467-023-41417-0)
Supplement: Supplementary file 3 — Reporting Summary [file 41467_2023_41417_MOESM3_ESM.pdf]

nature portfolio

Corresponding author(s): Prof. Dr. Meinrad Gawaz

Last updated by author(s): Aug 19, 2023

Reporting Summary

Nature Portfolio wishes to improve the reproducibility of the work that we publish. This form provides structure for consistency and transparency in reporting. For further information on Nature Portfolio policies, see our Editorial Policies and the Editorial Policy Checklist.

Statistics

For all statistical analyses, confirm that the following items are present in the figure legend, table legend, main text, or Methods section.

n/a	Confirmed	
[ ]	[x]	The exact sample size (*n*) for each experimental group/condition, given as a discrete number and unit of measurement
[ ]	[x]	A statement on whether measurements were taken from distinct samples or whether the same sample was measured repeatedly
[ ]	[x]	The statistical test(s) used AND whether they are one- or two-sided<br>*Only common tests should be described solely by name; describe more complex techniques in the Methods section.*
[x]	[ ]	A description of all covariates tested
[ ]	[x]	A description of any assumptions or corrections, such as tests of normality and adjustment for multiple comparisons
[ ]	[x]	A full description of the statistical parameters including central tendency (e.g. means) or other basic estimates (e.g. regression coefficient) AND variation (e.g. standard deviation) or associated estimates of uncertainty (e.g. confidence intervals)
[ ]	[x]	For null hypothesis testing, the test statistic (e.g. *F*, *t*, *r*) with confidence intervals, effect sizes, degrees of freedom and *P* value noted<br>*Give P values as exact values whenever suitable.*
[x]	[ ]	For Bayesian analysis, information on the choice of priors and Markov chain Monte Carlo settings
[ ]	[x]	For hierarchical and complex designs, identification of the appropriate level for tests and full reporting of outcomes
[ ]	[x]	Estimates of effect sizes (e.g. Cohen's *d*, Pearson's *r*), indicating how they were calculated

Our web collection on statistics for biologists contains articles on many of the points above.

Software and code

Policy information about availability of computer code

Data collection	Control FIVE (Witec, Ulm, Germany) Software was used for Raman image aquisition. Raw spectral data were preprocessed by cosmic ray removal, background subtraction and baseline correction with Project FIVE (Witec). Images of histological stainings were aquired using Nikon NIS-Elemens AR 5.21.00 (Nikon, Tokyo, Japan). Multicolor immunofluorescence images were generated with the MACSima Software v0.15.0 on a MACSima imaging system (Miltenyi Biotec, Bergisch Gladbach, Germany). Overlaying Raman scan images and MALDI or immunofluorescence images was perfomed with GIMP 2.10.8 (The GIMP Development Team, https://www.gimp.org). Antibody-positive pixels were selected with ImagesJ's Multi-point tool (ImageJ, National Institutes of Health, USA).
Data analysis	Data analysis was performed with R 4.0.3 on RStudio 1.3.1093 (Rstudio, Boston, Massachusetts, USA) and the following libraries: Seurat (4.0.2), SeuratObject (4.0.4), SeuratWrappers (0.3.0), BayesSpace (1.6.0), Monocle2 (2.18.0), Monocle3 (1.0.0), Rtsne (0.16), scater (1.19.9), pheatmap (1.0.12), RColorBrewer (1.1-2), data.table (1.14.2), factorMineR(2.5), factoextra(1.0.7), ggstats(0.9.4), cowplot (1.1.1), abind (1.4-5), assertthat (0.2.1), beeswarm (0.4.0), Cardinal (2.8.0), cellranger (1.1.0), cluster (2.1.2), clustree (0.5.0), data.table(1.14.0), dbplyr(2.1.1), DDRTree(0.1.5), densityCluster(0.3), dplyr(1.0.7), EBImage(4.32.0), edgeR(3.32.1), fastICA (1.2-3), fastmap(1.1.0), flashClust(1.01-2), FNN (1.1.3), forcats (0.5.1), ggaluvial (0.12.3), ggbeeswarm (0.6.0), ggforce (0.3.3), ggplot2 (3.3.5), ggpmisc(0.4.5), ggpubr (0.4.0), ggrepel (0.9.1), gridExtra (2.3), leiden(0.3.9), magrittr (2.0.1), Matrix (1.3-4), matrixStats(0.60.0), mclust (5.4.7), htmltools (0.5.2), moonBook (0.3.1), purrr (0.3.4), RColorBrewer (1.1-2), reshape2 (1.4.4), reticulate (1.22), rJava (1.0-4), rlang(0.4.11), rstatix(0.7.0), Rtsne (0.15), scater (1.18.6), scran (1.18.7), sctransform (0.3.3), scuttle (1.0.4), SparesM (1.81), sparseMatrixStats(1.2.1), sparsevd(0.2), spatstat.core (2.3-2), spatstat.data (2.1-2), spatstat.geom (2.3-1), spatstat.sparse (2.1-0), spatstat.utils(2.3-0),statmod(1.4.36), SummarizedExperiment (1.20.0), tibble(3.1.3.), tidygraph(1.2.0), tidyr(1.1.3), tidyselect(1.1.1), tidyverse(1.3.1), venneuler (1.1-3), xtable(1.8-4), XVector (0.30.0), zoo (1.8-9).<br>Multicolor immunofluorescence image analysis was done using the MACS® iQ View image analysis software version 1.1.1 (Miltenyi Biotec, Bergisch Gladbach, Germany).<br>Supervised machine learning was performed using Jupyter Notebook running Python 3.10.6 and the Python packages Matplotlib (3.7.1),

Numpy (1.22.0), Scipy (1.10.1), Tensorflow (2.12.0)

For manuscripts utilizing custom algorithms or software that are central to the research but not yet described in published literature, software must be made available to editors and reviewers. We strongly encourage code deposition in a community repository (e.g. GitHub). See the Nature Portfolio guidelines for submitting code & software for further information.

Data

Policy information about availability of data

All manuscripts must include a data availability statement. This statement should provide the following information, where applicable:

- Accession codes, unique identifiers, or web links for publicly available datasets
- A description of any restrictions on data availability
- For clinical datasets or third party data, please ensure that the statement adheres to our policy

Raw data from Raman analysis as well as images from histological stainings used in this article can be accessed online in this Zenodo Repository: https://doi.org/10.5281/zenodo.8265653. Source data are provided with this paper. Additional data can be obtained from the corresponding author upon request.

Research involving human participants, their data, or biological material

Policy information about studies with human participants or human data. See also policy information about sex, gender (identity/presentation), and sexual orientation and race, ethnicity and racism.

Reporting on sex and gender	N/A
Reporting on race, ethnicity, or other socially relevant groupings	N/A
Population characteristics	N/A
Recruitment	N/A
Ethics oversight	N/A

Note that full information on the approval of the study protocol must also be provided in the manuscript.

Field-specific reporting

Please select the one below that is the best fit for your research. If you are not sure, read the appropriate sections before making your selection.

[x] Life sciences [ ] Behavioural & social sciences [ ] Ecological, evolutionary & environmental sciences

For a reference copy of the document with all sections, see nature.com/documents/nr-reporting-summary-flat.pdf

Life sciences study design

All studies must disclose on these points even when the disclosure is negative.

Sample size	Exploratory analysis with no sample size calculation; sample sizes are indicated in each figure and/or legend and/or methods.
Data exclusions	If not stated otherwise, no data were excluded from analysis. As stated in the manuscript, data was subsetted to whole spectrum/fingerprint spectrum for analysis purpose, outliers defined as extreme values were filtered out and paraffin peaks were also removed as stated in the manuscript.
Replication	Results were replicated as shown in the Supplementary Material.
Randomization	Mice were randomly assigned to their treatment group.
Blinding	Data analysis was performed in an unsupervised manner. If applicable blinding to treatment/operation group was used.

Reporting for specific materials, systems and methods

We require information from authors about some types of materials, experimental systems and methods used in many studies. Here, indicate whether each material, system or method listed is relevant to your study. If you are not sure if a list item applies to your research, read the appropriate section before selecting a response.

Materials & experimental systems

n/a	Involved in the study
☐	☒ Antibodies
☒	☐ Eukaryotic cell lines
☒	☐ Palaeontology and archaeology
☐	☒ Animals and other organisms
☒	☐ Clinical data
☒	☐ Dual use research of concern
☒	☐ Plants

Methods

n/a	Involved in the study
☒	☐ ChIP-seq
☒	☐ Flow cytometry
☒	☐ MRI-based neuroimaging

Antibodies

Antibodies used

All antibodies used are listed in Supplementary Table S2:
Target/Reagent - Clone - Fluorochrome - Dilution - Order No. - Supplier
Alpha-Smooth Muscle Actin - 1A4 Alexa Fluor™ 488 - 1:50 - 53-9760-82 - Thermo Fisher Scientific
Beta Actin - REAL1032 - PE - 1:50 - 130-127-405 - Miltenyi Biotec
CD105 - MJ7-18 - FITC - 1:50 - 130-102-915 - Miltenyi Biotec
CD11b - M1-70-15-11-5 - FITC - 1:50 - 130-113-796 - Miltenyi Biotec
CD11c - N418 - FITC - 1:50 - 130-122-939 - Miltenyi Biotec
CD2 - REA959 - FITC - 1:50 - 130-115-958 - Miltenyi Biotec
CD3 - REA641 - FITC - 1:50 - 130-119-798 - Miltenyi Biotec
CD31 - REAL260 - PE - 1:50 - 130-118-936 - Miltenyi Biotec
CD4 - REA604 - PE - 1:50 - 130-116-509 - Miltenyi Biotec
CD41 - REA1194 - PE - 1:50 - 130-122-760 - Miltenyi Biotec
CD44 - REA664 - PE - 1:50 - 130-118-694 - Miltenyi Biotec
CD45 - REA737 - FITC - 1:50 - 130-110-796 - Miltenyi Biotec
CD61 - REA1192 - PE - 1:50 - 130-122-148 - Miltenyi Biotec
CD68 - REA835 - FITC - 1:50 - 130-112-855 - Miltenyi Biotec
CD80 - REA983 - FITC - 1:50 - 130-116-459 - Miltenyi Biotec
CD8b REA793 - FITC - 1:50 - 130-111-710 - Miltenyi Biotec
Cardiac Troponin T - REA400 - PE - 1:50 - 130-120-405 - Miltenyi Biotec
Cytokeratin 19 A-3 - PE - 1:50 - sc376126PE - Santa Cruz Biotechnology
DAPI Staining Solution - DAPI - 1:100 - 130-111-570 - Miltenyi Biotec
F4/80 - REA126 - FITC - 1:50 - 130-117-509 - Miltenyi Biotec
Ki67 - REA183 - FITC - 1:50 - 130-117-691 - Miltenyi Biotec
Ly-6G - REA526 - FITC - 1:50 - 130-120-820 - Miltenyi Biotec
MHC Class II - REA813 - FITC - 1:50 - 130-112-386 - Miltenyi Biotec
Vimentin - E-5 - FITC - 1:200 - sc-373717 - FITC - Santa Cruz Biotechnology

Validation

Antibodies were chosen based on published IHC compatibility and on validations for MICS by the vendors. Internal validation was additionally performed previously by our collaborators on various murine tissues (liver, spleen, muscle). Furthermore, controls were included in the individual experiments of this publication (murine spleen).

Animals and other research organisms

Policy information about studies involving animals; ARRIVE guidelines recommended for reporting animal research, and Sex and Gender in Research

Laboratory animals

For the hypertrophy mouse model we utilized ApoE knockout mice (B6.129P2-Apoetm1Unc/J) purchased from Charles River (Boston, Massachusetts, USA). For the myocardial infarction mouse model we used wildtype mice of the Pf4Cre Ackr3 knockout strain (B6.Cg-Thy1a-(Ackr3)Ackr3tm1Fma-Tg(Pf4-cre)Q3Rsko/J) from the Jackson Laboratories (Bar Harbor, Maine, USA).
Mice were housed under specific pathogen-free conditions at the University of Tuebingen. Mice between 8 and 12 weeks of age were involved in animal experiments.

Wild animals

N/A

Reporting on sex

both male and female mice were used

Field-collected samples

N/A

Ethics oversight

All animal procedures were performed according to the German animal protection law and approved by the local authorities (Regierungspräsidium Tübingen, TVA M5/17, M20/15, M04/19G, M02/20G and M01/21G). To reduce the number of experimental animals, tissue samples were reused from experiments included within other projects. The data gained in this novel analysis method is genuine and therefore no data is published elsewhere.

Note that full information on the approval of the study protocol must also be provided in the manuscript.
